# SoyMAGIC: An Unprecedented Platform for Genetic Studies and Breeding Activities in Soybean

**DOI:** 10.3389/fpls.2022.945471

**Published:** 2022-07-07

**Authors:** Seyed Mohammad Hashemi, Gregory Perry, Istvan Rajcan, Milad Eskandari

**Affiliations:** Department of Plant Agriculture, Ontario Agriculture College, University of Guelph, Guelph, ON, Canada

**Keywords:** soybean (*Glycine max* L.), genetic linkage map, genotyping by sequencing, multi-parent advanced generation inter-crosses, Seed composition/quality

## Abstract

Multi-Parent Advanced Generation Inter-Cross (MAGIC) populations are emerging genetic platforms for high-resolution and fine mapping of quantitative traits, such as agronomic and seed composition traits in soybean (*Glycine max* L.). We have established an eight-parent MAGIC population, comprising 721 recombinant inbred lines (RILs), through conical inter-mating of eight soybean lines. The parental lines were genetically diverse elite cultivars carrying different agronomic and seed composition characteristics, including amino acids and fatty acids, as well as oil and protein concentrations. This study aimed to introduce soybean MAGIC (SoyMAGIC) population as an unprecedented platform for genotypic and phenotypic investigation of agronomic and seed quality traits in soybean. The RILs were evaluated for important seed composition traits using replicated field trials during 2020 and 2021. To measure the seed composition traits, near-infrared reflectance (NIR) was employed. The RILs were genotyped using genotyping-by-sequencing (GBS) method to decipher the genome and discover single-nucleotide polymorphic (SNP) markers among the RILs. A high-density linkage map was constructed through inclusive composite interval mapping (ICIM). The linkage map was 3,770.75 cM in length and contained 12,007 SNP markers. Chromosomes 11 and 18 were recorded as the shortest and longest linkage groups with 71.01 and 341.15 cM in length, respectively. Observed transgressive segregation of the selected traits and higher recombination frequency across the genome confirmed the capability of MAGIC population in reshuffling the diversity in the soybean genome among the RILs. The assessment of haplotype blocks indicated an uneven distribution of the parents’ genomes in RILs, suggesting cryptic influence against or in favor of certain parental genomes. The SoyMAGIC population is a recombined genetic material that will accelerate further genomic studies and the development of soybean cultivars with improved seed quality traits through the development and implementation of reliable molecular-based toolkits.

## Introduction

Since the 1920s, soybean [*Glycine max* (L.) Merr.] has been one of the major sources of protein and oil for human food and livestock feed in Canada (Singh and Hymowitz, 1999). Demand for this “king of beans” has been steadily increasing year-over-year due to its nutritional values for human and livestock, as well as industrial applications (Thrane et al., 2017). This growing demand has created a significant market for varieties with increased seed quality and yield, along with a range of improved agronomic traits. However, one of the main challenges for soybean breeders is the complexity associated with accumulating many of the desired quantitative traits in new cultivars. Many of these traits are regulated by multiple genes, located in different genomic regions, and tend to be dynamically regulated by a range of environmental, molecular, and biochemical factors (Whiting et al., 2020). A crucial step toward overcoming this challenge is deciphering the genetic structure of these quantitative traits, which can provide a prospect for plant breeders on how to select and develop cultivars with accumulated required traits.

Producing genetically recombinant crops through crossing two genetically diverse parents, so-called bi-parental crosses, has been one of the most important and common approaches for genetic studies and cultivar developments by plant geneticists and breeders. Genetic variation of the parental lines provides the opportunity to decipher and map genomic regions, quantitative trait loci (QTL), which are associated with the trait of interest (Miles et al., 2008). A wide range of genetic studies have been conducted to date to identify QTL regions associated with soybean seed quality traits using bi-parental populations (Eskandari et al., 2013; Pei et al., 2018; Chen et al., 2021). Nevertheless, bi-parental populations despite having strong mapping power suffer from insufficiency of recombination events and genetic diversity for a given locus, which results from genetic segregation of loci coming from only two parents (Diouf and Pascual, 2021). In addition, in respect to soybean seed quality traits, as each QTL has a smaller effect on the trait (Diers et al., 1992; Hu et al., 2021), achieving higher mapping resolution, i.e., “fine mapping,” for developing more durable molecular markers, can be challenging using this type of populations.

To address these limitations, various strategies have been proposed, including Advanced Intercrossed Lines (AILs), and Genome-Wide Association Studies (GWAS; Darvasi and Soller, 1995; Ozaki et al., 2002). However, AILs suffer from a low degree of genetic variation as a result of the presence of only two parents, and GWAS efficiency is also limited because of undetermined pedigree, missing parental information, and obtaining some false positive responses (Tam et al., 2019). A novel approach called “Multi-parent Advanced Generation Inter Crosses (MAGIC),” which was introduced by Kover et al. (2009), can to some extent address the above issues. In this approach, MAGIC populations resolve the issues associated with bi-parental analyses, and have a greater overall power in terms of genetic diversity, population structure, and mapping resolution (Huang et al., 2015; Diouf and Pascual, 2021). Developing MAGIC populations in self-pollinated crops includes crossing multiple genetically diverse inbred parental lines for several cycles, followed by single-seed descent selection process to produce recombinant inbred lines (RILs) carrying a mosaic of genome blocks from all parents (Scott et al., 2020). So far, the successful establishment of MAGIC population has been presented for several strategic crops such as maize (Jiménez-Galindo et al., 2019), barley (Novakazi et al., 2020), rice (Ponce et al., 2018), soybean (Shivakumar et al., 2018), and wheat (Stadlmeier et al., 2018). Scientific research publications in which MAGIC populations are used as the platform is showing a 250% increase in the last 10 years (Diouf and Pascual, 2021). The latter is facilitated by cost-effective, continuing, and reliable advances in high-throughput genotyping and phenotyping technologies that facilitated the establishment and evaluation of MAGIC populations with a large number of RILs along with well-developed phenotypic datasets.

The objective of this study was to develop and establish Soy MAGIC, an 8-founder soybean MAGIC population carrying various agronomic and seed composition traits, which can be used by researchers as an everlasting platform for deciphering and fine mapping of QTL associated with their target traits, and also to develop new value-added cultivars. Here, we present the process of SoyMAGIC development, high-density genetic linkage map construction as well as genetic features and validation of the population as a new genetic tool in soybean. The SoyMAGIC population with hundreds of RILs, each with a unique genetic combination of the eight parents and phenotypic performance, delivers a broad genetic resource for improving genetic gains of important traits in breeding programs as well as allowing for high precision QTL mapping of complex traits in soybean.

## Materials and Methods

### Development of Soybean MAGIC Population

**Figure 1:**
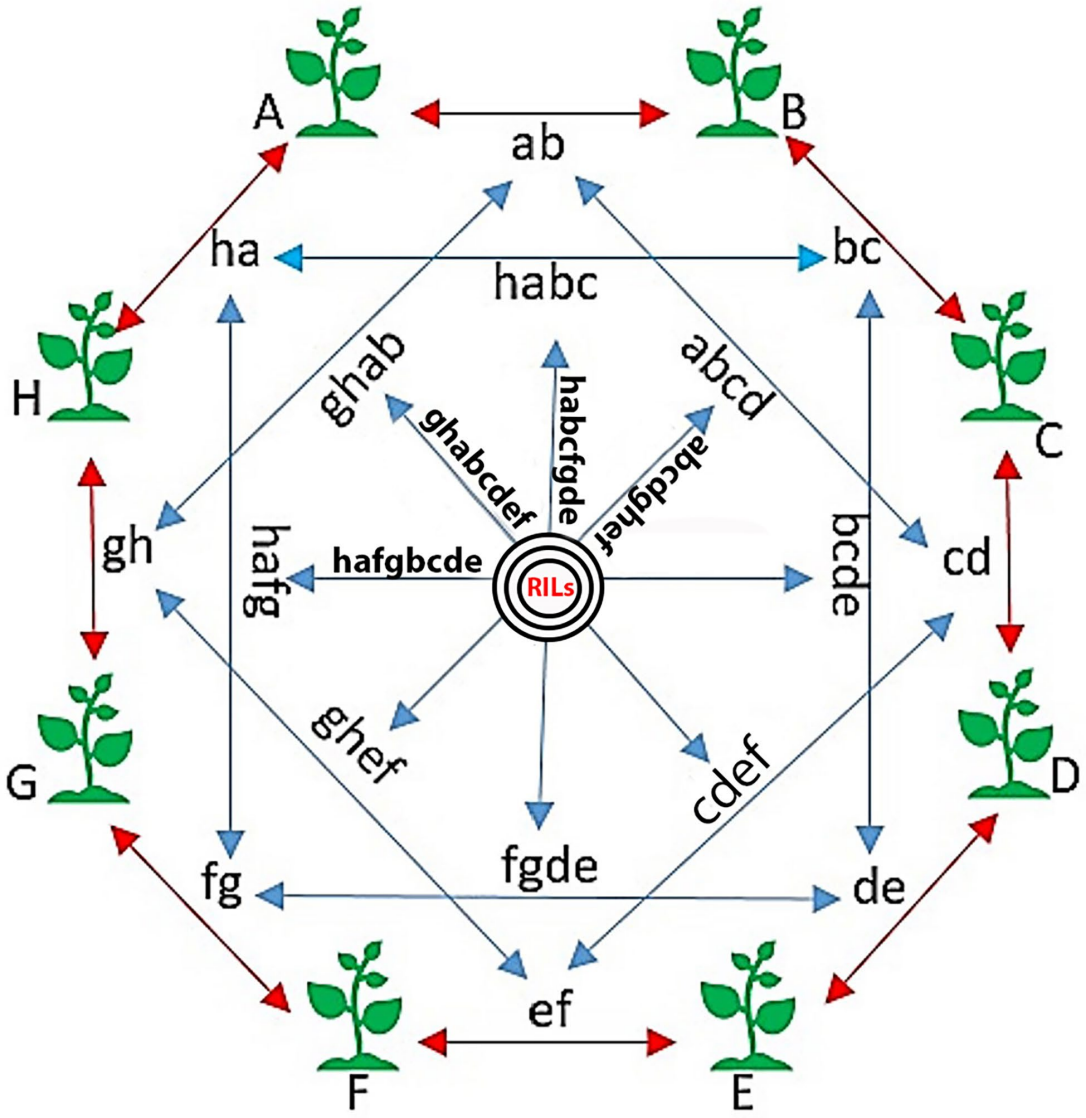
The conical cross used to establish the SoyMAGIC population. Capital words are representing eight elite parental cultivars, (A) OAC Prosper, (B) OAC 13-55C-HL, (C) OAC 07-78C-LL, (D) AC X790P, (E) RG 46, (F) RG 22, (G) RG 11, and (H) RG 23. Two-way crosses are represented by lower case letters (ab, bc, cd, de, ef, fg, gh, and ha). Four-way crosses are represented by four lowercase letters (abcd, bcde, bcde, fgde, ghef, hafg, ghab, and habc). Eight-way crosses are represented by eight lowercase letters (bcdeghab, fgdehabc, ghefabcd, hafgbcde, etc.). Black circles are showing the selfing generations, which ends up to the final RILs.

To develop the SoyMAGIC population, the following eight elite soybean lines were used as the founders: (A) OAC Prosper (Eskandari et al., 2017), (B) OAC 13-55C-HL, (C) OAC 07-78C-LL, (D) AC X790P (Poysa and Buzzell, 2001), (E) RG 46, (F) RG 22, (G) RG 11, and (H) RG 23 (Figure 1). These genetically diverse parental lines were selected based on their diverse phenotypic performance for important agronomic and seed quality traits (Table 1). Parental lines were inter-crossed in the form of conical crosses, consisting of eight parents and three cycles of crosses (Figure 1). In the first cycle, for each cross, the F_1_ seeds of eight 2-way mating combinations of the eight parents were generated in a way that each parent was used once as the female parent and once as the male parent. In the second cycle, F_1_ seeds of eight 4-way crosses, executed between the 2-way F_1_ plants, were generated such that each founding parent is present only once as the female and once as the male. Following the same pattern, the F_1_ seeds of eight 8-way crosses were generated by crossing the 4-way F_1_ plants. The plants resulting from the advanced inter-crossing stage were progressed four generations by single seed decent (SSD) to create 721 homozygous recombinant inbreed individuals.

**Table 1:** Descriptive characteristics of the parental lines for establishing the SoyMAGIC population.

Parent ID	Parent name	Comment/characteristic
A	OAC Prosper	High protein, high yield, large seed, semi-determinate
B	OAC 13-55C-HL	High protein, high yield, and high linoleic
C	OAC 07-78C-LL	High protein, high yield, and low linoleic
D	AC X790P	Average yield, very high protein, large seed size
E	RG 46	High protein and oleic acid
F	RG 22	High oil, low stearic, and high linoleic acids
G	RG 11	High oil, high oleic, and low linolenic acids
H	RG 23	High protein, low stearic, and high oleic acids

### Experimental Design and Phenotyping

The RIL population was propagated in Ridgetown, Ontario, Canada (42°26′55.32″ N, 81°52′41.49″ W), during 2020 and 2021. The experiment was set up as a randomized complete block design (RCBD) with nearest neighbor adjustment and two replicates. Each plot consisted of five rows, 4.2 m long, with a row spacing of 43 cm. The rows were trimmed to 3.8 m in length after emergence, and the inside three rows were harvested. In each plot, 500 soybean seeds were planted to reach a plant density of 54 seeds per square meter (m^−1^). The plots were managed using conventional standard tillage, standard pest, and weed management treatments. Plants in three middle rows were harvested after reaching full maturity.

The total chemical composition of soybean seed (30 g) was measured using Perten DA 7250 SD Near-Infrared Reflectance (NIR) spectrometer (Perten Instruments, Hägersten, Sweden). Seed samples were placed in a 9 mm diameter clear glass bottle at 4 mm height for the NIR spectrometer. Evaluation of seeds was performed for chemical components concentration as intact (without any treatment) using calibrations provided by Perten Instruments, as reported by Whiting et al. (2020). Three technical replications were applied for each measurement. Statistical analysis and visualization of the phenotype data were completed using R software packages including ggplot2, heatmaply, pastecs, and plotly.

### DNA Extraction and High-Throughput Genotyping

Young leaves were collected from each individual RILs and parental lines and stored at −80° C after lyophilization. Afterward, DNA was extracted using the Macherey-Nagle NuceloSpin II DNA kit (MACHEREY-NAGEL, Germany) according to the manufacturer’s instructions. DNA quality and quantity were assessed through Nano-drop spectrophotometer ND-1000 (Nanodrop Technologies, Inc., Wilmington, DE, United States) along with a Qubit v2.0 Fluorometer (Thermo Fisher Scientific Inc., United States), respectively. DNA quality of parental lines was verified using 1% agarose gel (Voltage) and stained with ethidium bromide prior to imaging on a GelDoc system (Supplementary Figure S1).

To genotype the RILs, sequencing libraries were prepared based on the genotyping by sequencing (GBS) protocol as explained by Elshire et al. (2011) except for the use of selective primers, which is described by Sonah et al. (2013) at the Plateforme d’analyses ge’nomiques (IBIS, Universite´ Laval). Normalized DNA concentrations of 10 ng/ml and restriction endonuclease of “*Ape*KI” were used in library preparation. Parental lines were genotyped by whole genome sequencing to obtain comprehensive genetic information as well as enough material for further investigations. Sequencing reads of parental lines were aligned to the reference genome, “William 82.” For the RILs, the variant call format (VCF) file was filtered out *via* VCFtools.^1^ After removing markers with more than 80% missing rate 183,482 SNPs remained out of 2,797,528 SNP markers. After individual level filtering, out of 760 individuals, 721 remained. Only bi-allelic SNPs remained. SNP imputation for the missing genotypes was carried out based on the haplotype structure of parental lines.

Physical map investigation and visualization were completed using rMVP and ggplot2 packages, R software (Wickham, 2017; Yin et al., 2021). Allelic contribution of parental lines in each chromosome was measured using “calc.genoprob” function with an error probability of 0.01 in the qtl2 package, R software (Broman et al., 2019).

### Population Structure

Principal component analysis (PCA) was carried out using TASSEL V5.2 to calculate the patterns of multi-locus variation (Bradbury et al., 2007). To illustrate the dispersion of the RILs in the population, the first two principal components (PCs) were used. According to the method of (Nei and Li, 1979), pairwise similarity coefficients were determined for all pairwise combinations of the RILs. To explore and visualized the familial relatedness among RILs, a Kinship matrix was also calculated using Genome Associated Prediction Integrated Tool (GAPIT) package in R (Lipka et al., 2012; Supplementary Figures S2 and S3).

### Construction of Genetic Linkage Map

Genetic linkage map constriction of SoyMAGIC population was conducted using the inclusive composite interval mapping (ICIM-ADD) method in GAPL V1.2 software (Zhang et al., 2019). Before running the map construction, quality of the genotypic data was checked by the software. First, “SNP data conversion” function was used to convert the genetic dataset to the format of the software. Non-polymorphed markers either in parents or progenies and markers which were missing in one or more parents were filtered out. Afterward, identification and filtering of redundant markers was applied to remove the markers with a missing rate of ≥10%, while the markers with the minimum missing rate were set to present the co-localized markers. In a particular population, a set of co-localized markers was defined as one bin. Markers with heterozygosity of more than 12.5% were discarded.

“Map construction in multi-parent derived pure-line populations” function was used to construct the genetic linkage map of SoyMAGIC population. Anchoring of markers with known chromosome ID on the physical map was the first step. Then, a grouping of markers was accomplished through anchored marker information and a threshold of marker recombination frequency (REC) of 0.3 for unanchored markers. For marker ordering, the two-optTSP and nearest-neighbor algorithms were used (Lin and Kernighen, 1973). Eventually, a window size of five-SNP was used as the rippling standard to measure the sum of adjacent recombination frequencies. Kosambi’s mapping function was used to convert the recombination frequency into map distance and the visualization of the genetic map was carried out using LinkageMapView package in R software (Ouellette et al., 2018).

## Results

### Population Development and Genotyping

**Figure 2:**
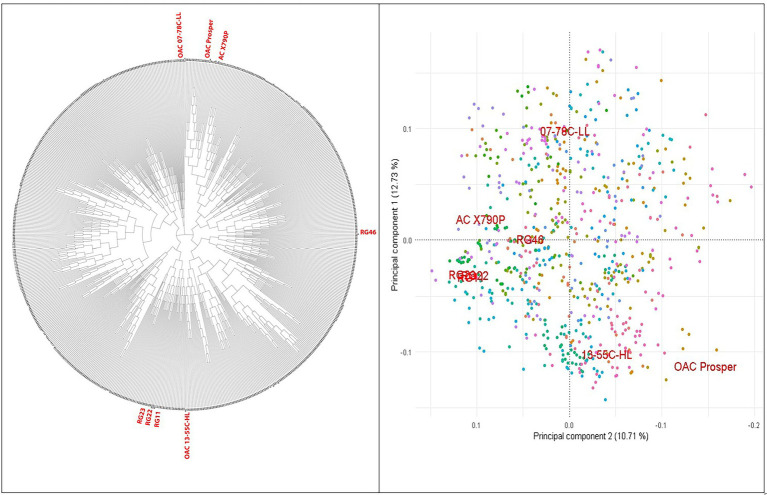
PCA and phylogenetic relationships of the 716 SoyMAGIC RILs and eight parental lines (in red) based on 122747 SNP markers.

A set of 721 soybean MAGIC RILs was produced through three and four generations of advanced inter-crossing and self-pollination, respectively (Figure 1). GBS of RILs resulted in a total of 183,342 SNPs that were polymorphic between the eight parents and RILs. The RILs were on average 87.9% homozygous and appeared highly diverse and clustered uniformly relative to their eight parents, among which RG11, RG22, and RG23 were closer to each other than the other parent-to-parent relationships (Figure 2).

### Genomic Features and Recombination Frequency of SoyMAGIC

**Figure 3:**
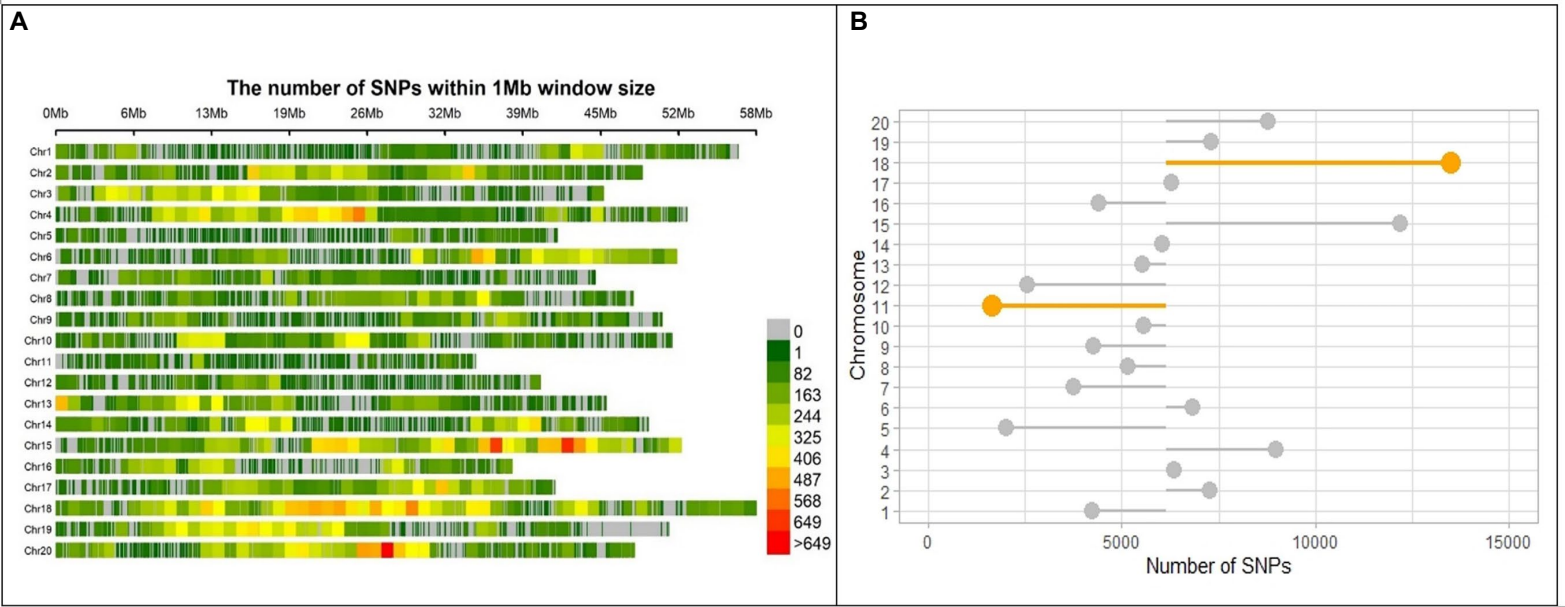
SNP marker distribution on the genome of SoyMAGIC RILs. **(A)** Genome-wide distribution of SNP markers in the RILs of soybean MAGIC population. The number of SNPs is calculated and visualized in 1 Mb window size for each of the chromosomes (Chr). The number of markers per Mb is color-coded. **(B)** Number of SNP markers for each chromosome. The mean number of SNPs, 6317, across the whole genome was used as a baseline for intra-chromosome comparisons. Chromosomes 18 and 11 with highest and lowest number of SNPs are highlighted, respectively.

After discarding markers with a MAF ≤0.05 and heterozygous rate ≤0.13 from the 183,342 polymorphic SNPs and 721 individuals, 716 individuals with 122,747 SNPs remained, which were distributed across the whole soybean genome with an average spacing of 0.915 kb. Marker distribution varied among and within 20 chromosomes of soybean (Figure 3A). In the physical map, the largest and smallest numbers of markers were observed in Chromosomes 18 and 11 with 13,476 and 1,644 SNPs, respectively (Figure 3B). The mean genome-wide SNP number was recorded as 6,317 per chromosome (Figure 3B). Comparison of detected chromosome-wide markers with a gene density of *G. max* cultivar “William 82, genome assembly version 4” (Schmutz et al., 2010) demonstrated higher SNP frequency in the centromeric region of chromosome 2, 4, 18, and 20.^2^

**Figure 4:**
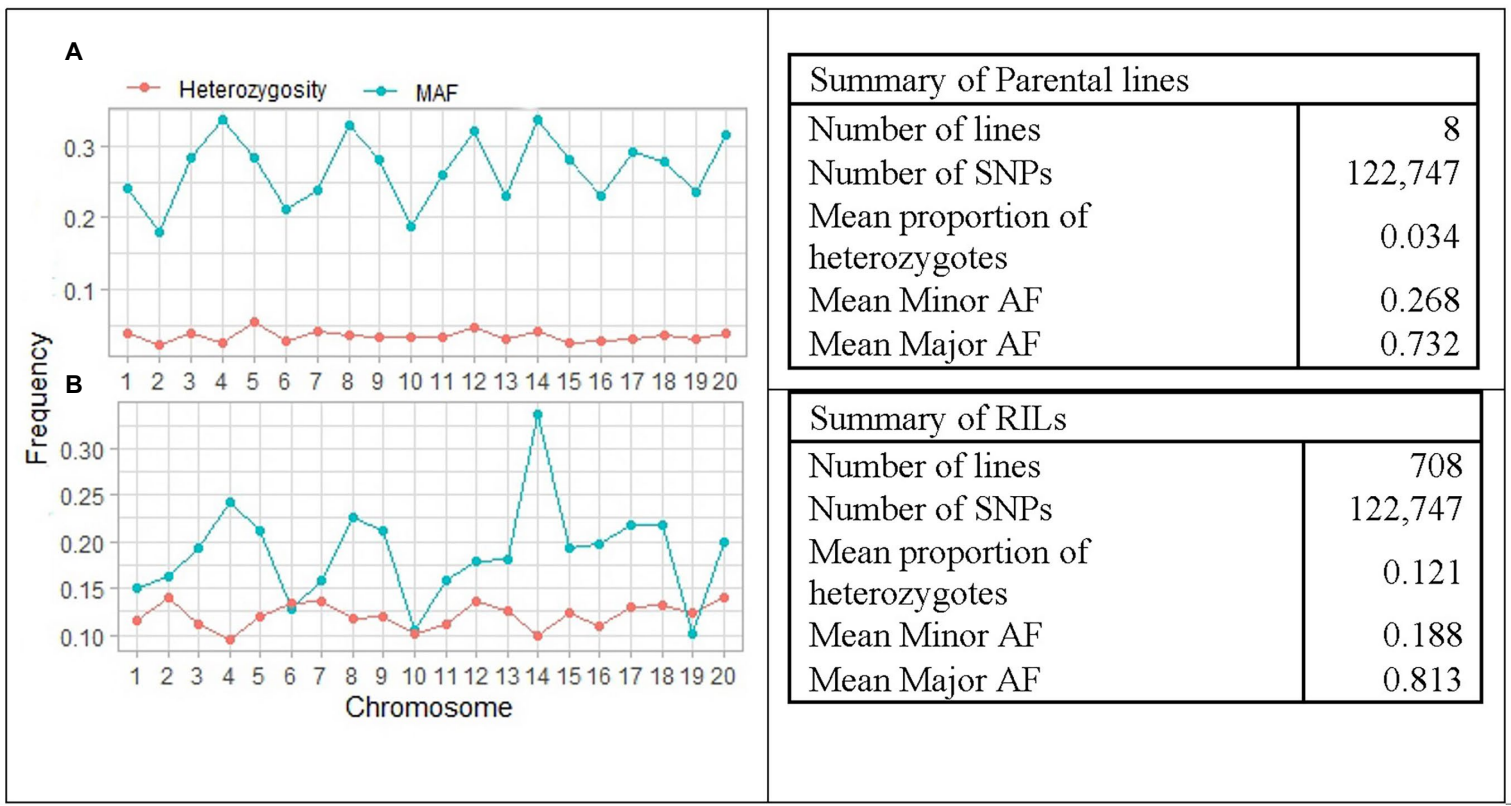
Summary and pattern of genetic features in RILs and parental lines of SoyMAGIC population after filtering out of low-quality SNPs. **(A)** and **(B)** display chromosome-wide distribution of minor allele frequency and mean proportion of heterozygosity in the SoyMAGIC parental lines and RILs, respectively. Summary statistic tables describe genome-wide proportion of heterozygosity and frequency of major and minor alleles of SoyMAGIC population in parental lines and RILs.

The distribution of average major allele frequency (AF), minor AF, and proportion of heterozygotes is illustrated in Figure 4. The average proportion of heterozygotes was 0.121 and 0.034 in the RILs and the parental lines, respectively. Average minor AF was 0.268 in parental lines and 0.188 among RILs, while the average major allele frequency was 0.732 and 0.812 in parental lines and RILs, respectively. The results indicated that the average MAF of the RILs was ranged from 0.101 on chromosome 19 to 0.337 on chromosome 14. This suggests that the SoyMAGIC RILs have higher average MAF and adequate polymorphism than the threshold (MAF < 0.05) for further genomic studies.

**Figure 5:**
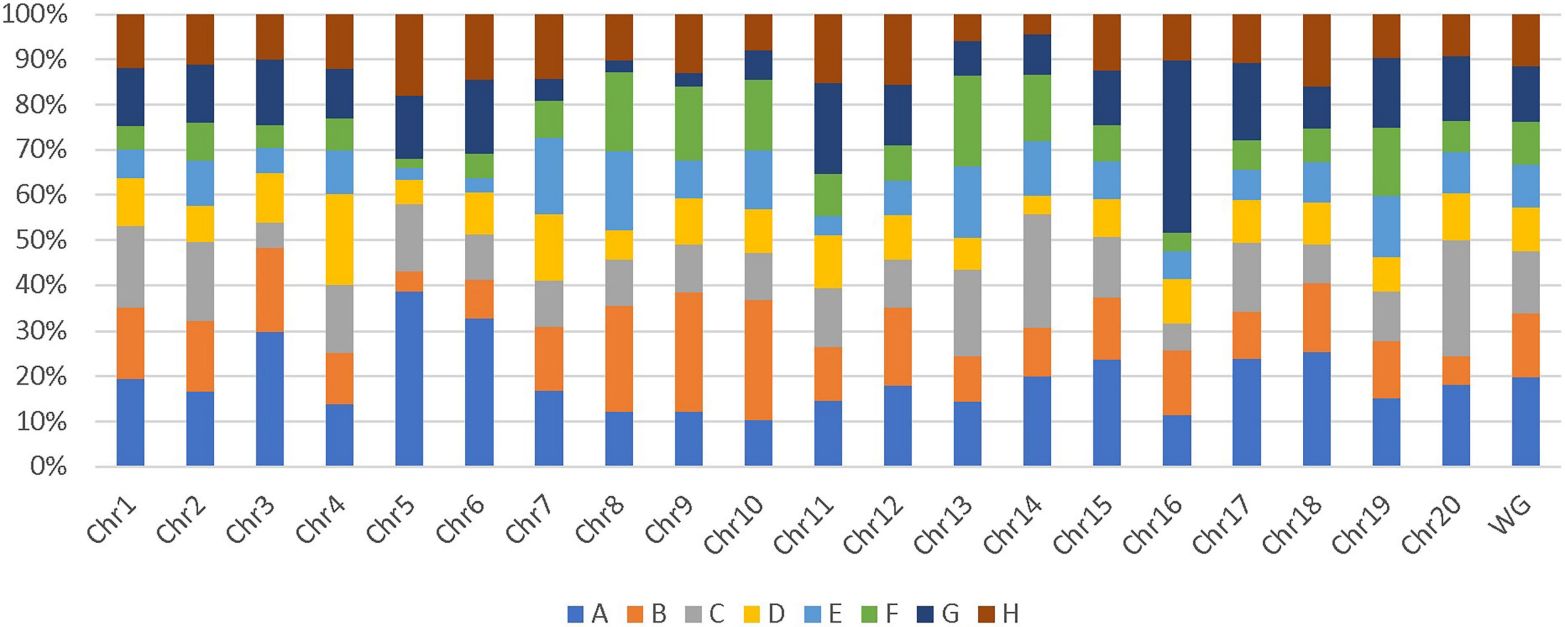
Chromosome-wide and genome-wide allele contribution of parental lines. WG represents the contribution of parental lines in whole genome.

Additionally, genome-wide and chromosome-wide assessment of parent’s allelic probability suggested that some parents contributed more to the SoyMAGIC RILs than others. Parents A and B with an average contribution of 19.3% and 14.2%, respectively, were more influential than the others (Figure 5). In contrast, parents D and E with an average contribution of 9.6% and 9.3%, respectively, were the least influential ones. Chromosomes 5 and 15 were recorded as the most unbalanced chromosomes with a maximum representation of parents A and G, respectively, and a minimum representation of parent F in both chromosomes.

### Phenotypic Variation in SoyMAGIC

**Figure 6:**
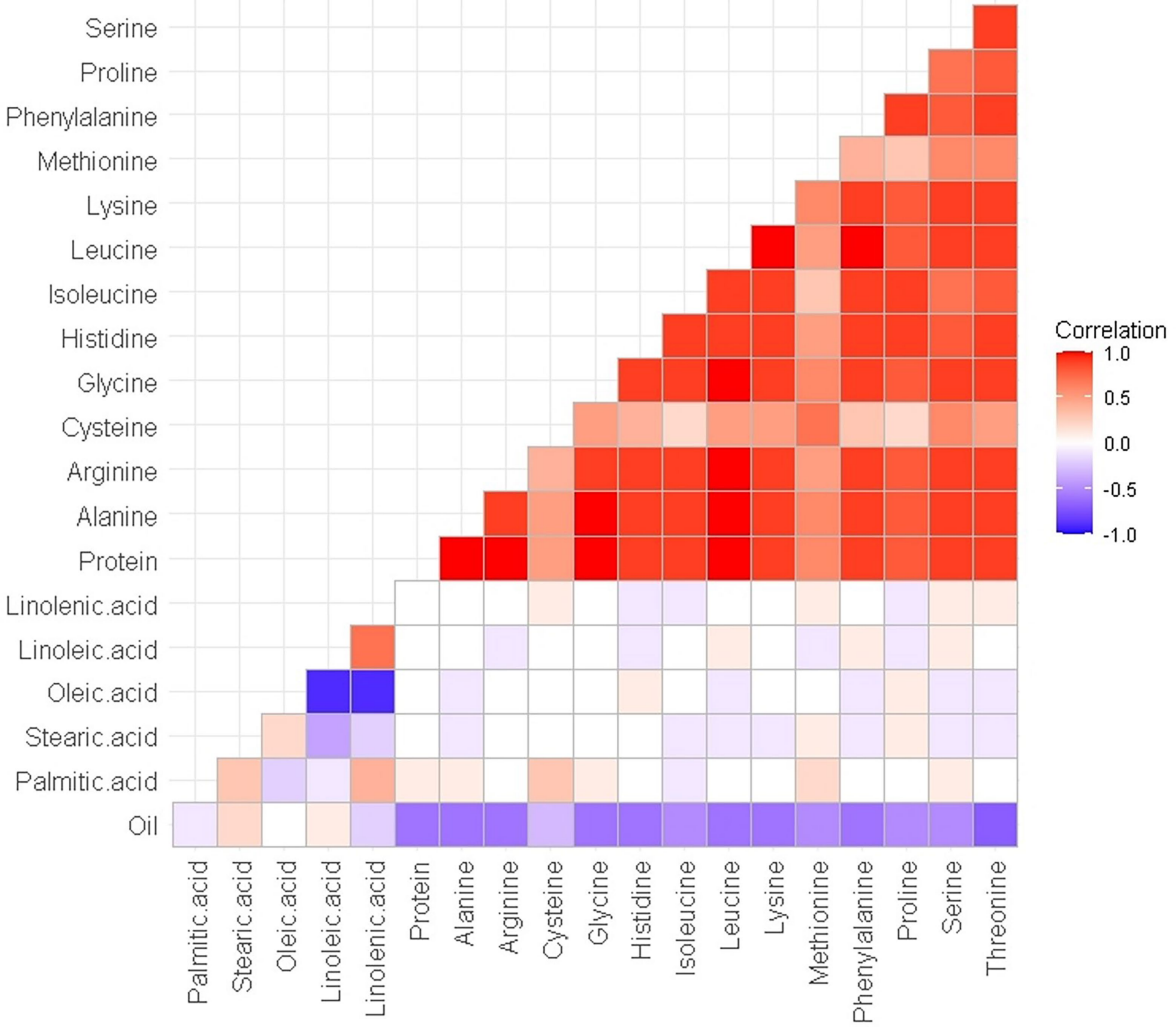
Pearson’s (*r*) correlation coefficient among seed quality traits in RILs of SoyMAGIC population.

The normal distribution of phenotypic data was verified and confirmed by Shapiro Wilk test after removing outliers. As illustrated in Table 2, descriptive statistics of phenotypic data for RILs and parental lines were calculated. Almost all the selected seed composition traits showed lower minimums and higher maximums for RILs than parental lines. Moreover, the mean value of the protein and oil concentration was recorded higher in RILs than in parental lines. In terms of the fatty acids, the mean value of oleic, palmitic, and stearic acids decreased, whereas the mean value of linolenic and linoleic acids increased in RILs as compared to the parental lines. Amino acids such as histidine, alanine, tryptophan, phenylalanine, tyrosine, and proline had higher mean values, whereas others had a lower mean for the RILs than the parental lines. Pearson’s correlation coefficient analysis of the seed quality traits was also measured among both parental lines and RILs. A positive correlation between all measured amino acids and seed protein concentration (*r* > 0.9) was observed. However, negative correlation was observed between the amino acids and fatty acids. In addition, as was expected, oleic acid showed a significant negative correlation with linoleic and linolenic acids (Figure 6).

**Table 2:** Quantitative statistics for seed composition traits of parents and RILs in SoyMAGIC population.

Traits	RILs				Parents			
	Min	Max	Mean	SD	Min	Max	Mean	SD
Protein	38.35	49.49	44.16	1.424	41.27	48.39	43.91	1.998
Oil	18.45	24.47	21.55	0.958	18.20	23.27	21.24	1.453
Palmitic acid	9.96	14.02	12.14	0.491	11.54	12.89	12.33	0.443
Stearic acid	2.80	5.45	4.14	0.364	3.37	5.54	4.40	0.651
Oleic acid	15.10	40.34	26.72	3.543	17.75	39.61	27.77	6.053
Linoleic acid	39.18	57.56	48.56	2.862	37.31	53.98	47.63	5.331
Linolenic acid	3.42	10.49	6.79	0.942	3.83	9.06	6.54	1.484
Alanine	1.66	2.02	1.85	0.045	1.75	1.98	1.83	0.064
Arginine	2.78	3.81	3.33	0.138	2.96	3.61	3.27	0.183
Cysteine	0.44	0.63	0.53	0.033	0.52	0.61	0.56	0.034
Glycine	1.67	2.05	1.85	0.051	1.77	2.01	1.85	0.071
Histidine	1.02	1.27	1.14	0.035	1.06	1.25	1.13	0.054
Isoleucine	1.82	2.23	2.06	0.056	1.92	2.19	2.01	0.081
Leucine	2.98	3.76	3.41	0.101	3.18	3.66	3.37	0.139
Lysine	2.28	3.02	2.76	0.078	2.52	2.98	2.73	0.122
Methionine	0.47	0.60	0.54	0.019	0.52	0.59	0.56	0.021
Phenylalanine	1.99	2.54	2.29	0.071	2.12	2.49	2.25	0.107
Proline	1.87	2.33	2.12	0.067	1.96	2.28	2.08	0.101
Serine	1.63	2.10	1.88	0.071	1.75	2.08	1.87	0.091
Threonine	1.46	1.76	1.60	0.044	1.51	1.73	1.59	0.065

### Genetic Linkage Map

After filtering out missing and low-quality markers using GAPL V1.2, 12,007 polymorphic SNPs were grouped into 20 linkage groups (LGs) with a total genome size of 3,770.75 centiMorgans (cM; Table 3). The highest and lowest map length was observed in LG18 and LG12 with 341.15 and 71.01 cM, respectively. The average length, across the LGs, was 188.54 cM. The number of markers for each linkage group ranged from 237 to 1,422 with an average of 600.35 marker. Additionally, the average marker interval was 0.37 cM. LG4 with an average distance of 0.15 cM was recorded as the densest LG, whereas LG7 had the largest average interval distance of 0.60 cM. The maximum and minimum interval distances were observed in LG19 and LG20 with 20.03 and 2.57 cM, respectively.

**Table 3:** Information on linkage map of the SoyMAGIC population.

Linkage group	Map length	No. of markers	No. of bins	Interval distance	
				Max	Average
1	151.08	377	110	6.17	0.4
2	165.7	421	116	3.76	0.39
3	180.18	709	215	5.55	0.25
4	212.03	1,422	484	6.03	0.15
5	153.77	391	131	4.18	0.39
6	215.75	464	167	5.4	0.46
7	180.69	303	95	5.67	0.6
8	182.65	496	163	6.55	0.37
9	273.47	655	229	5.8	0.42
10	183.13	352	113	7.62	0.52
11	137.45	266	98	5.7	0.52
12	71.01	247	77	3.09	0.29
13	179.05	381	143	4.79	0.47
14	263.07	1,158	493	7.52	0.23
15	230.97	1,110	378	5.24	0.21
16	203.09	609	233	7.12	0.33
17	189.05	593	199	6.54	0.32
18	341.15	1,126	322	5.65	0.3
19	137.37	237	63	20.03	0.58
20	120.1	690	232	2.57	0.17
Total	3770.75	12,007	4,061	20.03	0.37

## Discussion

MAGIC populations are exceptional genetic resources for improving the recombination frequency of resultant RILs and discovering marker-trait relationships with high accuracy and resolution accordingly (Scott et al., 2020). Multiple parents with greater phenotypic and genetic variation, as well as multiple rounds of inter-crossing and selfing, enhance the number of recombination events and therefore maximize mapping accuracy (Huang et al., 2015). Through inter-crossing diverse parents for a particular trait, the genetic variability in the final RILs increases, which is a decisive advantage of developing these types of populations for genetic studies (Scott et al., 2020). Several studies have previously exploited MAGIC populations for investigating genetic control of important trait in strategic crops such as maize (Jiménez-Galindo et al., 2019), rice (Ponce et al., 2018) and wheat (Stadlmeier et al., 2018). Here, we report the establishment of a soybean MAGIC (SoyMAGIC) population developed by combining eight parental lines that were genetically and phenotypically diverse for several agronomic and seed quality traits (Table 1, Supplementary Table S1, and Figure 1).

In plant breeding programs, a large population size is one of the necessary factors to maximize the mapping resolution (Beavis, 1998; Rosenthal and Borschbach, 2014). The SoyMAGIC population was maintained reasonably large at 721 RILs, to accumulate a wider range of recombination events, using a reciprocal conical design (Figure 1). To capture the maternal cytoplasmic genetic variance of parents (Morgan, 2013), the reciprocal conical crossing strategy was used during population development.

Soybean seed compositions, particularly oil and protein concentrations, are among the most studied traits in soybean due to their economic importance in the food and feed industries (Kumawat et al., 2016). Phenotypically, larger standard deviations, maximum and minimum values of the selected traits of RILs compared to parental lines (Table 2), confirmed the transgressive segregations and indicated the capability of SoyMAGIC population in reshuffling the genome in RILs. In fact, intensification of the genetic variation across the genome of RILs was because of the way that the population is developed. Similar results were reported for multi-parent populations of other plant crops such as rice, maize, cowpea, and eggplant, confirming the competence of multi-parent populations in reshuffling of genome and improving the recombination level (Dell’Acqua et al., 2015; Huynh et al., 2018; Ponce et al., 2020; Mangino et al., 2022). Since the eight parents were all completely inbred lines, the plants in each F_1_ set were homogeneously heterozygous. Theoretically, the F_1_s resulting from the four-way crosses, on the other hand, segregate and show substantial heterogeneity (Figure 1). This heterozygosity and heterogeneity generated individuals with recombined genotypes and phenotypes. Furthermore, using four generations of SSD selection, in which we did not apply any targeted selective pressure for any of the target traits, a genetically and phenotypically diverse RIL population consisting of 721 was generated and established as the SoyMAGIC population.

To discriminate genotypes for their genetic diversity in plant genetic and breeding activities, GBS has already been confirmed to be an exceptionally efficient and cost-effective approach for the genotyping of large multi and bi-parental populations (He et al., 2014; Kishor et al., 2021). WGS of parental lines has also been reported as a highly effective genotyping strategy in multiparent plant breeding programs, which can be employed in further genetic investigations such as QTL mapping and identification of candidate genes (Islam et al., 2016; Thyssen et al., 2019). Detection of 183,342 SNP markers across the genome, confirmed that GBS of RILs, imputed using WGS of the parental lines, could be a suitable method for generating a high-resolution map for soybean multiparent genotyping. In this study, higher number of SNP markers was observed around telomeric regions of most of the chromosomes, whereas chromosome 5, 7, 12 and 13 exhibited higher SNP density around centromeric area (Figure 3). These results reflect the strength of SoyMAGIC population in reshuffling alleles across the genome and providing a highly recombined genomic platform suitable for discovering QTL/candidate genes associated with complex traits. Theoretically, in an 8-parent MAGIC population, each of the parental lines should contribute 12.5%. However, certain paternal lines contributed more to the SoyMAGIC population than others (Figure 5). The observed variance in the contribution of founders might be caused by a variety of genotypic or environmental factors such as fertility reduction or male sterility due to environmental conditions (Brauner-Otto, 2014; Li et al., 2019).

**Figure 7:**
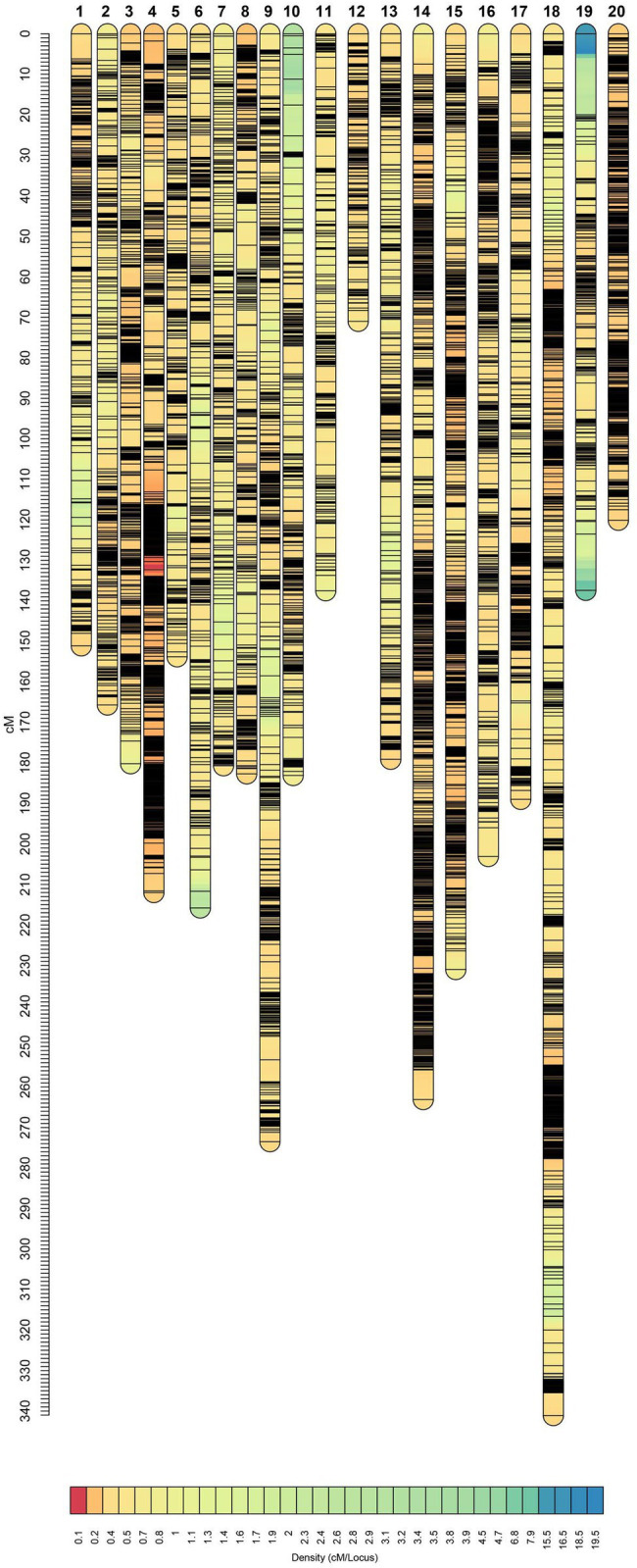
Genetic linkage map constructed from SoyMAGIC.

It has been shown that SNP discovery in soybeans is a challenging and time-consuming process (Wu et al., 2010). Limited sequence variation in currently cultivated varieties as well as the complicated nature of the soybean genome are two critical factors causing the complications (Choi et al., 2007). Considering these challenges, we have constructed a new and high-density genetic linkage map that contains 12,007 SNP markers with a genome length of 3,770.75 cM by employing an eight-parent RIL population. Compare to the previous studies on soybean genetic linkage maps of bi-parental populations (Hyten et al., 2010; Song et al., 2016), the current map demonstrated a greater number of distinct sites, comparable genome length, and shorter average bin size (Table 3, Figure 7). In comparison to bi-parental populations (Hyten et al., 2010), the SoyMAGIC population displayed a significantly higher number of marker alleles at each locus, which reflects the capacity of SoyMAGIC for enhancing genetic variation and recombination frequency in the population.

Establishing genetic linkage map is an important step for the dissection of genome regions associated with important agronomic and quality traits through identifying the location of quantitative trait loci (QTL; Williams, 2018). Through improving genetic recombination in RILs, SoyMAGIC has provided a desired platform for discovering marker’s location across the genome and constructing a high-density genetic linkage map, which, in turn, provided a strong platform for further marker-trait association investigations. So far, several MAGIC population-derived RILs have been developed to dissect the genome of many crops using different mapping strategies (Scott et al., 2020). For instance, Huynh et al. (2018) used linkage map in an eight parent cowpea MAGIC population with 305 RILs, leading to the successful detection of four QTL underlying flowering time. Huang et al. (2021) using genome-wide association mapping in an 8-way upland cotton MAGIC population, discovered 177 SNPs strongly associated with nine agronomic traits in multiple environments. SoyMAGIC population will provide researchers with immortal diverse plant materials that can be tested across a wide range of environments with different types of biotic and abiotic stresses for discovering environment-specific effects of genomic regions associated with traits. Genotypic and phenotypic data generated for these studies will be stored and made available to breeders for improving their selection criteria and establishing efficient breeding strategies.

## Conclusion

In addition to serving as an immortal genetic resource for precise marker-traits association studies and precise QTL mapping, SoyMAGIC will support breeding programs in the long run by offering valuable pre-breeding resources. The preliminary phenotypic data collected on agronomic and seed quality traits along with the SNP data set showed large phenotypic and genetic diversity among the lines within the population, which indicate the potential benefits and advantages of using this diverse germplasm in genetic studies and breeding activities by the soybean community. SoyMAGIC has been established by inter-crossing eight founders using reciprocal conical crosses in order to maintain maternal genetic materials and high recombination rate in the RILs. The population represents a valuable plant germplasm resource, which consists of 721 highly recombined RILs with a large degree of phenotypic variation. We have developed the first high-density genetic linkage map of an eight-parent MAGIC population in soybean that allows efficient discovery of gene-trait associations and QTL mapping of quantitatively inherited traits.

## Data Availability Statement

The original contributions presented in the study are publicly available. This data can be found here: https://github.com/SeyedMH/SoyMAGIC.

## Author Contributions

ME: conceptualization. SH: validation, data curation, visualization, and writing. SH and GP: formal analysis. SH, ME, IR, and GP: review and editing. ME and SH: project administration. All authors have read and agreed to the published version of the manuscript.

## Funding

This project was funded in part through the Ontario Regional Priorities Partnership Program (ON-RP3), a collaborative initiative between the Agricultural Adaptation Council, Ontario Genomics, the Government of Canada through Genome Canada, and SeCan.

## Conflict of Interest

The authors declare that the research was conducted in the absence of any commercial or financial relationships that could be construed as a potential conflict of interest.

## Publisher’s Note

All claims expressed in this article are solely those of the authors and do not necessarily represent those of their affiliated organizations, or those of the publisher, the editors and the reviewers. Any product that may be evaluated in this article, or claim that may be made by its manufacturer, is not guaranteed or endorsed by the publisher.

## Footnotes

^1^ https://vcftools.github.io/

^2^ https://www.soybase.org/gb2/gbrowse/glyma.Wm82.gnm4/
