# Supplementary material for: SoyMAGIC: An Unprecedented Platform for Genetic Studies and Breeding Activities in Soybean
Source: Front Plant Sci. 2022 Jul 7;13:945471. doi: 10.3389/fpls.2022.945471 (PMC9301248; doi:10.3389/fpls.2022.945471)
Supplement: Supplementary file 1 [file Data_Sheet_1.docx]

SoyMAGIC: a unique platform for genetic studies and breeding activities in soybean

Seyed Mohammad Hahsemi^1^, Gregory Perry^1^, Istvan Rajcan^1^, Milad Eskandari^1*^

^1^Department of Plant Agriculture, Ontario Agriculture College, University of Guelph, 50 Stone Road East, Guelph, ON N1G 2W1, Canada.

*** Correspondence:**Corresponding Author
[meskanda@uoguelph.ca](mailto:meskanda@uoguelph.ca)

Supplementary Material


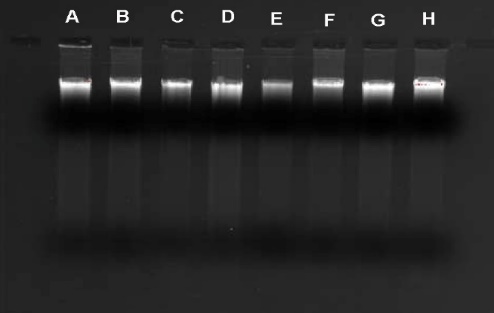


**Supplementary Figure 1.** Agarose gel (1%) electrophoresis of DNA from parental lines. Each lane (A to H) represents a parental line.


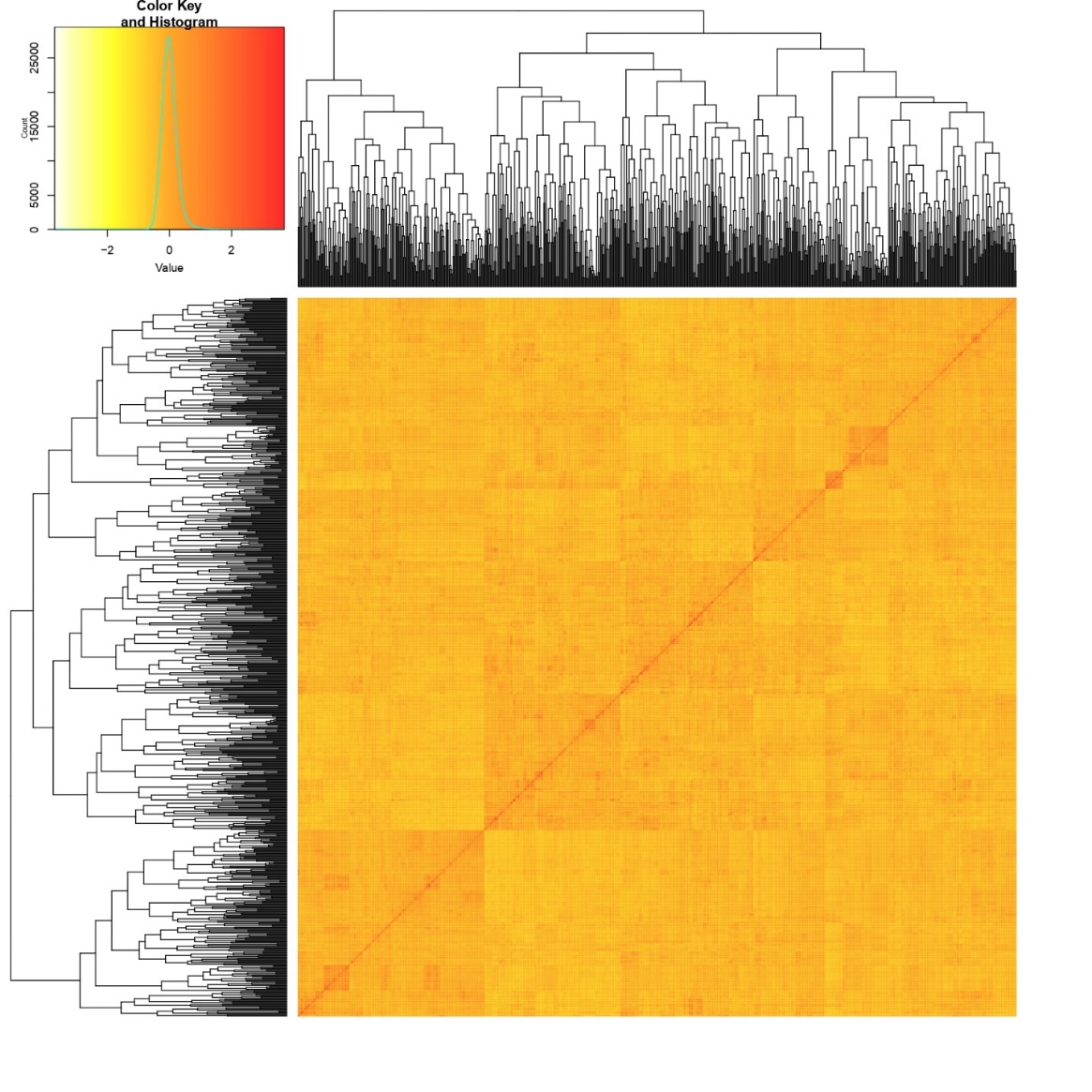


**Supplementary Figure 2.** Kinship matrix of SoyMAGIC displays relationship between RILs. The light colors represent less relatedness and dark colors represent more relatedness.


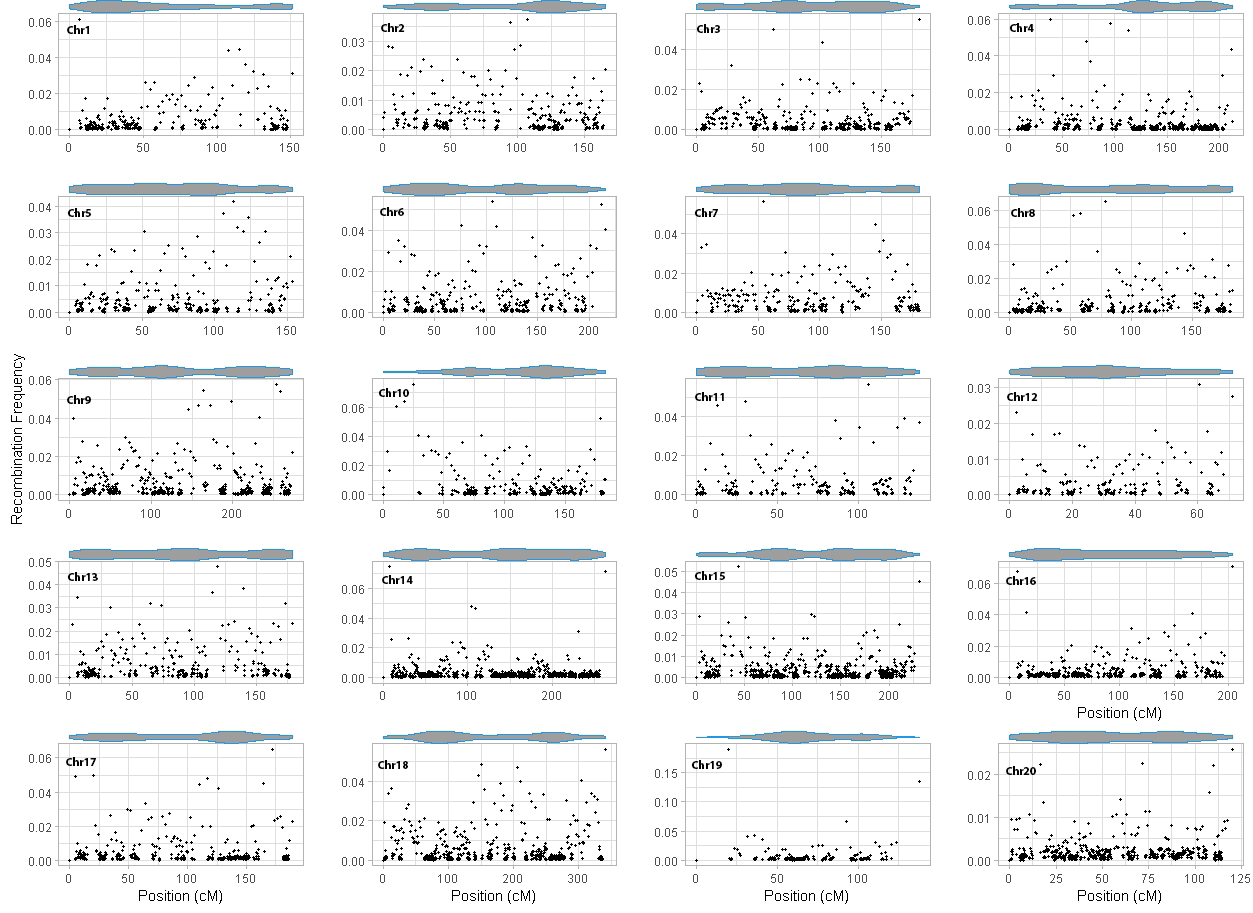


Supplementary Figure 3. Recombination frequency between two neighboring markers across 20 soybean chromosomes of SoyMAGIC population. Recombination frequency adjusted in range 0 to 1.

Supplementary Table 1. Additional phenotypic data of parents and RILs in SoyMAGIC population.

| Trait | Parents | RILs |
| --- | --- | --- |
| Flower color | 7 P: 1 W | 585 P: 123 W |
| Pubescence color | 6 T: 2 G | 539 T: 169 G |
| Hilum color | 5 Bl: 3 Y | 34 LtBr: 55 G: 28 DkBr: 310 Br: 239 Bl, Y:42 |

W: white, P: purple, T: **tawny, G: gray, LtBr: light brown, DkBr: dark brown, Br: brown, Bl: black, Y: yellow**
